# Exploration of chemotherapy-free regimen after multi-line chemotherapy-induced renal impairment in recurrent ovarian cancer: Case report and literature review

**DOI:** 10.3389/fonc.2022.1031045

**Published:** 2023-01-19

**Authors:** Liu-ping Zhang, Xiang Yang, Wei Zheng, Kai-xun Feng, Hu Li

**Affiliations:** Department of Gynecology, Guangzhou Panyu Central Hospital, Panyu Cancer Institute, Guangzhou, Guangdong, China

**Keywords:** recurrent ovarian cancer, renal impairment, chemotherapy-free regimen, niraparib, adenocarcinoma

## Abstract

**Introduction:**

Platinum-based combination chemotherapy is recommended first choice for relapsed ovarian cancer. However, many of the chemotherapeutic agents are nephrotoxic and can promote kidney dysfunction, which affect the efficacy of cancer treatment and the survival of the patient. There is a need to explore long-term treatments of chemotherapy-free regimen of chronic kidney disease in recurrent ovarian cancer.

**Case presentation:**

A 41-year-old female patient was presented with stage IIIC well-differentiated ovarian serous papillary adenocarcinoma in 2009. The patient had recurrence of platinum resistance after secondary cytoreductive surgery, and it was difficult to continue chemotherapy after multiple lines of chemotherapy due to myelosuppression, renal impairment and other factors. The patient accepted Niraparib-based treatment regimen after multi-line chemotherapy-induced stage 4 chronic kidney disease. Niraparib combined with anlotinib achieved median PFS of 11 months, disease re-progression, and the patient was switched to niraparib combined with letrozole from October 2021. No evidence of tumor progression was observed till date and the renal toxicity is acceptable.

**Conclusions:**

In patients with relapsed ovarian cancer, treatment becomes increasingly challenging to subsequent therapies because of renal impairment and emerging drug resistance. Niraparib-based treatment regimen may be a good choice for patients with well-differentiated serous adenocarcinoma of the ovary who are intolerant to chemotherapy.

## Introduction

Globally, 313,959 new ovarian cancer cases were estimated in 2020 of which 57,090 are from China (1, 2). High-grade serous ovarian cancer (HGSOC) is the most common histologic subtype, accounting for over 70% of ovarian cancer cases while low-grade serous ovarian cancer (LGSOC) represents about 10% cases (3). Despite maximal cytoreduction at the time of primary surgery and platinum-based adjuvant chemotherapy, 70-80% of patients with advanced ovarian cancer will relapse (4). Women with platinum-sensitive recurrent ovarian cancer (PSROC) can be treated with secondary cytoreductive surgery (SCS) and chemotherapy or chemotherapy alone. Systemic treatment is still the standard of care in relapsed ovarian cancer. Platinum-based combination chemotherapy is recommended first choice for relapsed patients. For platinum-resistant or platinum-sensitive ovarian cancer patients, non-platinum drugs such as doxorubicin, paclitaxel, gemcitabine, docetaxel and etoposide are recommended (5). However, renal impairment is a common serious complication of chemotherapy in ovarian cancer (6). A study reported acute kidney injury (AKI) in 10880 patients of 163,071 patients on systemic therapy for cancer with an cumulative incidence of 9.3% (7). A retrospective study reported AKI in 40.4% of patients (8). This indicates increased risk of AKI with systemic therapy for ovarian cancer which cannot be ignored. Hence, there is a need to explore new chemotherapy-free regimens for treating chemotherapy intolerant patients.

The emergence of poly (ADP-ribose) polymerase inhibitors (PARPi), antiangiogenics and immune checkpoint inhibitors (ICIs) have revolutionized the treatment of recurrent ovarian cancer. PARP plays a key role in the process of DNA damage repair. PARPi such as niraparib and olaparib, are approved by FDA as maintenance therapy for patients with PSROC and as posterior line therapy for patients with BRCA mutated recurrent ovarian cancer. NOVA study is the first study to report effectiveness of niraparib as maintenance therapy in patients with PSROC regardless of BRCA mutation (9). QUADRA study reported clinical benefit of niraparib in fourth or later line therapy among women with heavily pretreated ovarian cancer, especially in HRD-positive platinum-sensitive ovarian cancer (10).

Anlotinib, a tyrosine kinase inhibitor (TKI; inhibits tumor angiogenesis and growth) showed improvement in progression-free survival (PFS) and overall survival (OS) in patients with platinum-resistant or platinum-refractory ovarian cancer (11). Anti-angiogenic drugs induce hypoxia in tumors which in turn change DNA damage repair pathways, including homologous recombination (HR) pathway leading to unstable gene replication hence, increases the sensitivity of PARP inhibitors (12–14). Early clinical trials of anti-angiogenic inhibitors and PARP inhibitors suggest that this combination may bring good efficacy with less toxicity and side effects, and longer chemotherapy-free intervals.

Letrozole is a non-steroidal aromatase inhibitor (type II inhibitor) that inhibits estrogen signaling. Its key mechanism of action is the reversible competitive inhibition of aromatase without affecting the levels of other steroids (15). Women with LGSOC are typically diagnosed at a younger age and more likely to express estrogen and progesterone receptors. Hence, they may experience better survival outcomes with letrozole compared to women diagnosed with HGSC (16). Studies showed prolonged recurrence-free interval with letrozole in recurrent well-differentiated serous ovarian cancer, especially when used as maintenance therapy (17, 18). Biomarkers are essential for diagnosis of cancer. Cancer antigen 125 (CA-125) and human epididymis protein 4 (HE4) are the established biomarkers for detecting the recurrence. However, a study by Ferraro et al., founded that in many cases of OC follow-up there is a disagreement

between CA125 and HE4. Owing to renal impairment or different responses to treatment by two markers and HE4 estimations appeared affected by renal impairment. CA125 as most reliable biomarker for monitoring of ovarian cancer over HE4 (19).

To our knowledge, no case reports are published till date on exploratory treatment with niraparib-based chemotherapy-free regimens for patients with well-differentiated serous adenocarcinoma of the ovary who have severe renal impairment caused by prior chemotherapy. Here, we report a case of a 41-year-old female patient with well-differentiated serous papillary adenocarcinoma of the ovary. The patient had recurrence of platinum resistance after secondary cytoreduction, and it was difficult to continue chemotherapy after multiple lines of chemotherapy due to myelosuppression, renal impairment and other factors. Clinical benefit was observed from treatment with niraparib-based chemotherapy-free regimens with an acceptable safety profile till date.

## Case presentation

**Figure 1:**
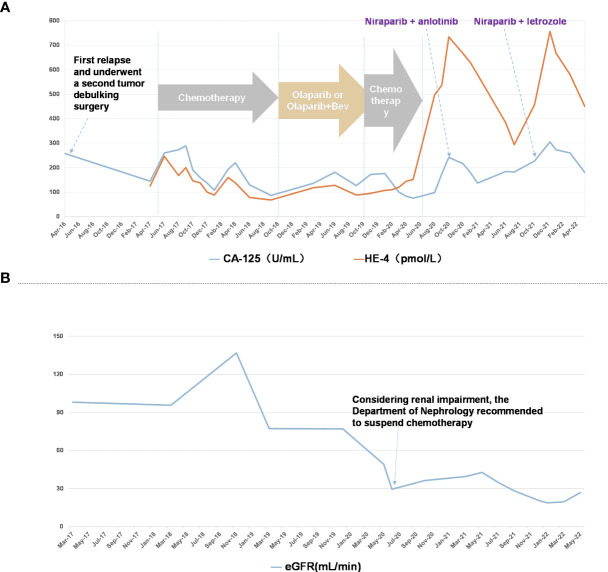
Patient course of disease progression and various treatment timelines **(A)** CA-125 (U/mL) and HE-4 (pmol/L) levels in patient through the timeline Apr 2016 to May 2022. **(B)** eGFR (mL/min) in patient through the timeline May 2017 to May 2021.

A 41-year-old female patient was presented with ovarian cyst assessed by physical examination was admitted to Guangzhou Panyu Central in August 2009. There was no dysmenorrhea, abdominal distension, abdominal pain, bowel habit change, nausea, and vomiting. The past medical, family and psychosocial history were unremarkable. In the bimanual gynecological examination, the uterus was normal in size and texture while an adnexal mass with indistinct margin can be felt. Transvaginal ultrasonography revealed tumor in the right ovary measuring 74×63 mm with a solid compartment. An elevated serum CA-125 levels (1428 U/mL [normal range, <35 U/mL]) were observed. The patient underwent ovarian cancer cytoreduction (R0; included hysterectomy, bilateral adnexectomy, omentectomy, resection of anterior bladder tumor and anterior rectal tumor, and dissection of pelvic lymph node) and received adjuvant platinum-based chemotherapy. Postoperative pathological findings were well-differentiated serous papillary adenocarcinoma of bilateral ovaries, invasion of cancer nests in the fibro-adipose tissue of the greater omentum, fibrous and adipose tissue of the bladder surface mass, prerectal mass, and fibrous and adipose tissue of the right external iliac lymph node with metastases. In April 2016 (6 years after the end of the first course of platinum-containing chemotherapy), CA-125 levels were elevated (258 U/mL), and CT imaging showed metastases of the abdomen. The patient underwent a second tumor debulking surgery in another hospital. Paclitaxel (210 mg) + carboplatin (450 mg) intravenous chemotherapy was administered for 6 cycles after the second cytoreduction (**Figure 1**). The patient’s CA-125 levels were descended to normal range after the chemotherapy (**Figure 1**). In April 2017 (one year after the second surgery) the patient had relapse again which was supported by the elevated levels of CA-125 (144 U/mL) and mediastinal retroperitoneal lymph node and right pleural recurrence were identified in the follow-up PET-CT scans. A biopsy of the right pleural nodule confirmed a metastasis of well-differentiated serous papillary adenocarcinoma of the ovary. The patient received different treatment regimens as summarized in **Table 1**.

**Table 1:** Different treatment regimens followed throughout the study.

Time	Treatment plan
29-05-2017 to 09-08-2017	Paclitaxel 210 mg + carboplatin 460 mg IV chemotherapy × 4 cycles
14-09-2017	Gemcitabine 1.5 g on d1, d8, d15 + cisplatin 110 mg IV chemotherapy
21-10-2017	Gemcitabine 1.2 g on d1, 1.0 g on d8 + cisplatin 80 mg IV chemotherapy
22-11-2017	Gemcitabine 1.0 g on d1, d8 + cisplatin 80 mg IV chemotherapy
Observed grade IV myelosuppression and persisted even after dose reduction	
27-12-2017	Docetaxel 120 mg + Cisplatin 80 mg IV chemotherapy
01-02-2018 to 28-02-2018	Docetaxel 115 mg + Cisplatin 115 mg IV chemotherapy
The tumor progressed rapidly, and the MDT discussion recommended genetic testing. The results showed no mutation in BRCA1 and BRCA2 while UGT1AI*6, UGT1A1*28, UGT1A1-3156 had gene polymorphism.	
04-2018 to 10-2018	Irinotecan 120 mg on d1, d8, d15 + bevacizumab 300 mg q3w IV × 7 cycles
The patient refused all intravenous chemotherapy regimens and requested targeted therapy	
01-2019 to 06-2019	Olaparib 300 mg po bid
14-06-2019 to 17-10-2019	Olaparib 300 mg po bid + bevacizumab 480mg IV × 5 cycles
The tumor continued to progress hence the intravenous chemotherapy is repeated	
15-11-2019 to 07-02-2020	Doxorubicin liposome 50 mg + bevacizumab 480 mg IV × 4 cycles
07-03-2020	Doxorubicin liposome 50 mg + cisplatin 120 mg + bevacizumab 480 mg IV ×1 cycle
08-04-2020 to 10-06-2020	Doxorubicin liposome 50 mg + cisplatin 120 mg intravenous chemotherapy × 3 cycles
In June 2020, a progressive increase in creatinine (creatinine value 182 μmmol/L) was observed. Considering renal impairment, the Department of Nephrology recommended to suspend chemotherapy.	
10-2020 to 04-2021	Niraparib 100 mg po qd + anlotinib 10 mg po qd
05-2021 to 10-2021	Niraparib 200 mg po qd + anlotinib 10 mg po qd + megestrol 160 mg
10-2021 to present	Niraparib 200 mg po qd + letrozole 2.5 mg

IV, intravenous; MDT, Multidisciplinary team meetings; po bid, orally twice daily; po qd, orally once a day.

**Figure 2:**
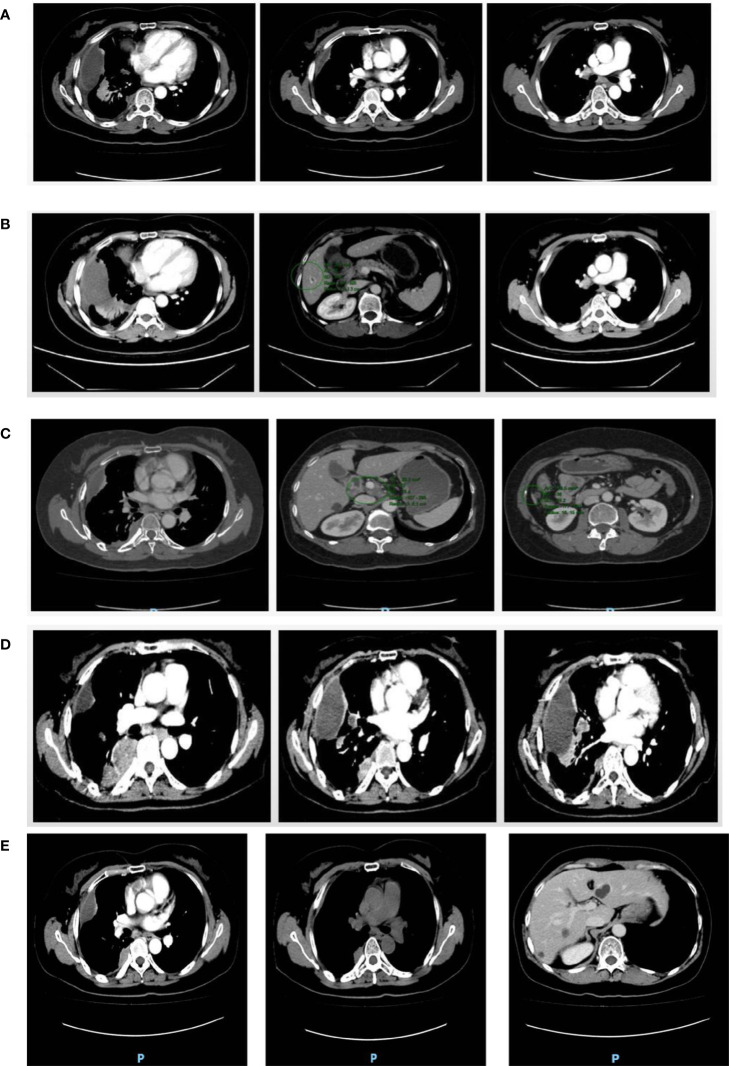
CT images. **(A)** Right pleura and interlobular pleura; **(B)** Right pleura and liver; **(C)** Right pleura; **(D)** Right pleura and right lung; **(E)** Right pleura and right lung and liver. **(A)** In December 2018, multiple small nodule foci were observed in the right pleura and interlobar pleura indicating the possibility of multiple metastases but smaller in size than earlier foci. **(B)** In October 2019, multiple metastases were observed in the right pleura, which were more and larger than previous lesions. The nodular foci were enhanced at the edge of the S6 segment of the liver. **(C)** In July 2020, multiple metastases in the right pleura were observed with no significant change in size and number compared with the previous lesions. A lymph node in the hilar region was observed swollen indicating lymph node metastasis. **(D)** In April 2021, multiple metastases that were larger in size and number were observed in the right pleura and right lung. Multiple enlarged lymph nodes were found in mediastinum and right hilar region. **(E)** In April 2022, the lesions in the right pleura and right lung were slightly smaller than before; the lesion in hepatic S6 segment was not significantly changed than before.

From May 2017 to November 2017, patient received combination of paclitaxel + carboplatin and gemcitabine + cisplatin in different doses, which caused grade IV myelosuppression that persisted even after dose reduction. Hence, the patient received docetaxel + cisplatin at different doses till February 2018. As the tumor progressed rapidly, genetic testing of tumor showed no mutation in BRCA1 and BRCA2 while UGT1AI*6, UGT1A1*28, UGT1A1-3156 gene polymorphism was detected. Further, irinotecan and bevacizumab were administered intravenously once in 3 weeks for 7 cycles from April 2018 to October 2018. With the patient’s request targeted therapy (olaparib alone or in combination with bevacizumab) was given from January 2019 to October 2019. As the tumor progressed even with targeted therapy, patient received doxorubicin, cisplatin and bevacizumab in different combinations and doses from November 2019 to June 2020. Post which a progressive increase in creatinine levels was observed indicating renal impairment (**Figure 1**), hence chemotherapy was discontinued. Since October 2020, patient received chemotherapy-free regimen (niraparib, anlotinib and megestrol). **Figure 2** represents magnetic resonance images (CT) of lungs and liver showing metastasis. After 11 months of PFS, disease re-progression, and the patient was switched to niraparib combined with letrozole from October 2021. By data cut-off (May 30, 2022), patient was still on treatment. Computed tomography (CT) showed no signs of recurrence.

## Discussion

In the present case report, a 41-year-old female patient with recurrent well-differentiated serous ovarian cancer developed platinum resistance after secondary cytoreduction. After multiple lines of chemotherapy, continuing chemotherapy further was found difficult due to myelosuppression, renal impairment and other factors. Niraparib combined with antiangiogenic drugs showed better PFS. Niraparib combined with letrozole was individually selected for further treatment as it was effective and less-toxic. It is suggested that PARPi combined with letrozole may have beneficial effect in patients with well-differentiated serous adenocarcinoma of the ovary who are intolerant to chemotherapy. The limitations associated with this case is that there are no clinical guideline or trails that predict the efficiency and toxicity of the combinations of niraparib and letrozole in CKD patients. Meanwhile, in targeted agents, there are many off-target and adverse effects that directly or indirectly affect the kidney. As presented in our case, niraparib combined with antiangiogenic drugs had lightly aggravated renal impairment.

Chemotherapy plays an important role in the treatment of gynecological malignant tumors. Most recurrent ovarian cancers require multiple courses of chemotherapy but patients tend to develop progressive drug resistance over time (20, 21). Most chemotherapeutic drugs and their metabolites are metabolized by the kidneys which can lead to renal damage. The nephrotoxicity of chemotherapy is usually related to the dose and repeated administration. Hence, accurate assessment of kidney function is crucial for informing decisions regarding anticancer drug dosing and eligibility. To our knowledge, the CKD-EPI equation is currently recommended for the assessment of kidney function in cancer patients by the Kidney Disease Improving Global Outcomes (KDIGO) guideline group (22, 23). Several studies have shown the accuracy and precision of the CKD-EPI equation in the cancer population over other methodologies (23, 24). In the present case, after multiple lines of chemotherapy, the patient was diagnosed with stage 4 CKD based on eGFRCr calculated by CKD-EPI even after dose reduction. Several studies have clearly demonstrated inferior outcomes with development of stage 4 or 5 CKD posing a challenge for promising curative therapeutic regimens (25, 26). A retrospective observational study has demonstrated that all-cause mortality and cancer-specific mortality were significantly higher in CKD patients than in non-CKD patients (27). It could be argued that kidney dysfunction creates an inflammatory microenvironment and oxidative stress, which can establish the ideal environment for cancer development (28). Presently, there are no standard recommended treatment regimens available to treat patients with chemotherapy-induced renal dysfunction.

Thus, creating a need for emergence of non-chemotherapeutic drugs that help in solving the problems of ovarian cancer drug resistance and chemotherapy intolerance. The aim of non-chemotherapy treatment as an alternative to chemotherapy is to save patients from the physical and psychological pain caused by chemotherapy and to follow the principles of effective, non-toxic and economical application. In recent years, significant progress has been made in targeted therapy of ovarian cancer. The emergence of PARPi and anti-angiogenesis inhibitors has added new anti-cancer “weapon” to chemotherapy treatment. AVANOVA2 study reported significant improvement in PFS with niraparib plus bevacizumab (11.9 months) compared to niraparib alone (5.5 months) in patients without BRCA mutations (29), suggesting combination of drugs with different mechanism of actions can achieve better results in patients with platinum-resistant or sensitive, chemotherapy intolerant recurrent ovarian cancer.

ANNIE study showed promising antitumor activity and tolerable safety with niraparib in combination with anlotinib with an objective response rate (ORR) of 50% (95% CI 33.8%-66.2%) and PFS of 8.3 months in patients with platinum-resistant recurrent ovarian cancer (30). In the present case, olaparib alone and in combination with bevacizumab failed to effectively control disease progression. Based on literature, niraparib was given in combination with anlotinib which led to a PFS of 11 months with acceptable safety. OreO/ENGOT Ov-38 study on maintenance olaparib rechallenge in patients with ovarian cancer previously treated with a PARPi reported significant improvement in PFS with maintenance olaparib compared to placebo. The subgroup analysis of the study showed a more pronounced benefit with different PARPi after re-progression (31). In the present case, tumor progression was observed even with olaparib + bevacizumab maintenance therapy hence the treatment was switched to niraparib + anlotinib which achieved median PFS of 11 months, suggesting PARPi combined with anti-angiogenesis drugs may benefit treatment. The key questions regarding overlap or combinations of antiangiogenics and PARPi should be addressed in future clinical trials.

Although PARPi and antiangiogenic drug therapy have increased the treatment options for recurrent ovarian cancer, options remain limited for rare histologic subtypes, such as well-differentiated serous ovarian cancer. Letrozole can be used as an option for recurrent ovarian cancer, and its clinical benefits were demonstrated in several phase II prospective trials (32–34). A retrospective study demonstrated that addition of letrozole as maintenance therapy in patients with ovarian cancer increased recurrent free survival by 60% vs 38.5% in the control group (35). In another phase-II study, patients with relapsed ovarian cancer treated with letrozole showed an ORR of 15% with acceptable safety profiles (36).

Furthermore, several attractive combination strategies were proposed to optimize responsiveness to letrozole. The strategies including letrozole combined with phosphoinositide 3-kinase (PI3K) inhibitors and mammalian target of rapamycin (mTOR) inhibitors, and the potential synergism has already been demonstrated in breast and endometrial cancers (37–40). On this basis, the combination therapy has been explored in recurrent ovarian cancer. The combination of everolimus and letrozole is associated with a promising 47% 12-week PFS rate in patients with ER-positive relapsed high-grade ovarian cancer with acceptable toxicity (41). The combination of ribociclib and letrozole assessed in a small phase II trial in ovarian and endometrial cancer demonstrated 55% and 50% 12-week PFS rates (42). Letrozole combined with niraparib was given to the patient of current study and no evidence of tumor progression was observed till date. The mechanism of action of the combination of PARPi and letrozole is not clear, but in this case, it did show a certain anti-tumor effect, and the safety of this regimen for patients with renal insufficiency is acceptable.

As far as we know, there are no published cases of exploratory treatment with niraparib-based chemotherapy regimens in patients with well-differentiated serous papillary adenocarcinoma of the ovary with severe renal damage caused by chemotherapy. However, as this a single case, large-scale clinical trials are warranted to further explore the efficacy and safety of PARPi in combination with letrozole in the future.

## Conclusion

For advanced platinum-resistant recurrent ovarian cancer after multi-line chemotherapy progression or intolerance due to chemotherapy toxicity, it may be a good choice to switch to antiangiogenics drugs combined with PARPi therapy. At the same time, if the patients progressed after the previous pre-sequence PARPi treatment, other types of PAPRi combined with anti-angiogenic drugs may still be beneficial. More importantly, the application of letrozole in combination with PARPi in low-grade serous adenocarcinoma of ovarian cancer should receive renewed attention.

## Patient perspective

Receiving chemotherapy was a painful process. During the chemotherapy, I always felt nausea, vomiting, constipation, anorexia, dysgeusia, fatigue and pain. It mainly affected my commitment to family and work. I prefer targeted therapy to chemotherapy for reasons related to convenience, home treatment, and needle avoidance. The process of anti-cancer is tormenting but hopeful.

## Data availability statement

The original contributions presented in the study are included in the article/supplementary material. Further inquiries can be directed to the corresponding author.

## Ethics statement

The studies involving human participants were reviewed and approved by NA. The patients/participants provided their written informed consent to participate in this study. Written informed consent was obtained from the individual(s) for the publication of any potentially identifiable images or data included in this article.

## Author contributions

All authors listed have made a substantial, direct, and intellectual contribution to the work and approved it for publication.
